# Curcumin protects ANIT-induced cholestasis through signaling pathway of FXR-regulated bile acid and inflammation

**DOI:** 10.1038/srep33052

**Published:** 2016-09-14

**Authors:** Fan Yang, Xiaowen Tang, Lili Ding, Yue zhou, Qiaoling Yang, Junting Gong, Guangyun Wang, Zhengtao Wang, Li Yang

**Affiliations:** 1 The MOE Key Laboratory for Standardization of Chinese Medicines and the Shanghai Key Laboratory of Compound Chinese Medicines Institute of Chinese Materia Medica, Shanghai University of Traditional Chinese Medicine, Shanghai 201203, China; 2 Center for Chinese Medical Therapy and Systems Biology, Shanghai University of Traditional Chinese Medicine, Shanghai 201203, China

## Abstract

Cholestasis is a clinically significant symptom and widely associated with liver diseases, however, there are very few effective therapies for cholestasis. Danning tablet (DNT, a Chinese patent medicine preparation) has been clinically used to treat human liver and gallbladder diseases for more than 20 years in China. However, which ingredients of DNT contributed to this beneficial effect and their mechanistic underpinnings have been largely unknown. In the present study, we discovered that DNT not only demonstrated greater benefits for cholecystitis patients after cholecystectomy surgery in clinic but also showed protective effect against alpha-naphthylisothiocyanate (ANIT)-induced cholestasis model in rodent. Curcumin, one major compound derived from DNT, exerted the protective effect against cholestasis through farnesoid X receptor (FXR), which has been focused as potential therapeutic targets for treating cholestasis. The underlying mechanism of curcumin against cholestasis was restoring bile acid homeostasis and antagonizing inflammatory responses in a FXR-dependent manner and in turn contributed to overall cholestasis attenuation. Collectively, curcumin can be served as a potential treatment option for liver injury with cholestasis.

Cholestasis is a common symptom of liver injuries and characterized as the interruption of bile flow from hepatocytes to intestine, which leads to bile acid accumulation in the liver, resulting in oxidative stress, inflammation, apoptosis and fibrosis. Cholestasis is highly associated with a wide spectrum of diseases, such as obstructive jaundice^1^, biliary atresia^2^, gallstones^3^, acute hepatitis^4^, cystic fibrosis^5^, primary sclerosing cholangitis (PSC)^6^ and primary biliary cirrhosis (PBC)^7^. So far, there are very few effective therapies for cholestasis, with ursodeoxycholic acid being the only approved drug by Food and Drug Administration^8^. Therefore, it is extremely important to develop new therapeutic medicines. In China, Danning tablet (DNT, a Chinese patented medicine preparation, Shanghai Hutchison Pharmaceuticals) has been clinically used to treat human liver and gallbladder disease for more than 20 years. We have previously discovered that DNT exerted anti-cholestatic effects in rodent liver injury models. However, the translational potential and mechanistic underpinnings are largely unknown.

The pathogenesis of cholestasis is intimately related to dysregulated bile acid homeostasis. Farnesoid X receptor (FXR) belongs to a nuclear receptor superfamily and functions as a key bile acid sensor. As a result, FXR may serve as a potential therapeutic target in cholestasis treatment. Indeed, previous studies have demonstrated that FXR agonist (GW4064) could improve cholestasis symptoms induced by alpha-naphthylisothiocyanate (ANIT) or bile duct ligation (BDL) in rodent models^9^. In addition, emerging evidence have implicated the pronounced activation of inflammatory responses in hepatic cell apoptosis and necrosis during cholestasis. However, whether bile acids overload triggers the inflammatory cascade in hepatocytes has not been characterized.

In this study, we showed that DNT exerted anti-cholestatic effects not only in rodents, but also in cholecystitis patients after cholecystectomy surgery. Mechanistically, curcumin derived from DNT was identified to play a predominant role in alleviating cholestasis-associated bile acid deregulation and consequent inflammation. Our data also unambiguously proved that FXR mediated the therapeutic effects of DNT and curcumin, thus supporting to further investigate FXR as a molecular target in cholestasis management.

## Results

### DNT reduced pathological bile acid accumulation and inflammation in cholecystitis patients and rodents

**Figure 1:**
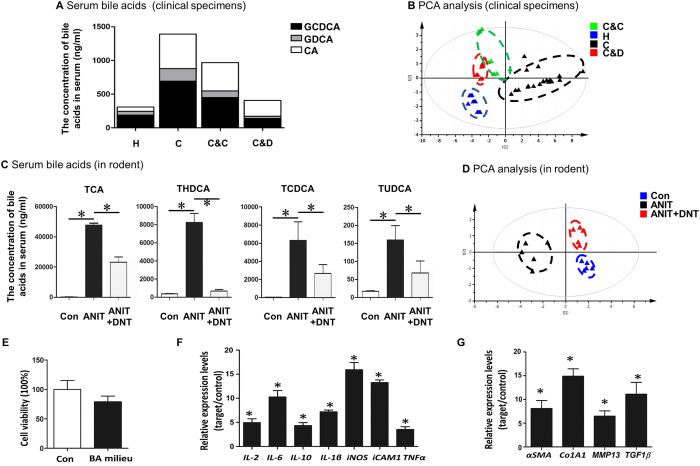
DNT reduced pathological bile acid accumulation and inflammation in cholecystitis patients and rodents. (**A,B**) Twenty patients with cholecystitis (represents C) and 10 healthy people (represents H) were participated in this study. They are double-blind randomized into two groups after cholecystectomy operation. The treatment groups received DNT regimen (represents C&D) for 12 weeks since 5 days after operation relative to the normal antibiotics therapy which control groups received (represents C&C). Their blood was collected at 13 weeks after the operation. All samples were prepared for detection of bile acid concentrations (**A**) and inflammatory cytokines (**B**). (**C,D**) ANIT-induced animal model was established to assess the protective effect of DNT in rodent. Serum levels of TCA, THDCA, TCDCA, and TUDCA were quantified by UPLC-MS (**C**) and hepatic inflammation-related genes were studied by Real-time PCR and visualized by PCA (**D**). (**E–G**) Primary mouse hepatocyte cells were exposure to bile acid milieu for 24 h. Cell viability was measured by CCK-8 assay (**E**). Inflammation-related genes, *IL-2*, *IL-6*, *IL-10*, *IL-1β*, *iNOS*, *iCAM1* and *TNFα* (**F**) as well as fibrosis-related genes, *αSMA*, *Co1A1*, *MMP13* and *TGF1β* (**G**) were detected by Real-time PCR. Data are presented as mean ± SD. *p < 0.05.

Since our previous work has revealed anti-cholestatic functions of Danning tablet (DNT) in rodent models^10^,^11^, we sought to validate these findings in clinic. To this end, we recruited 20 patients with cholecystitis based on the diagnostic results by biochemistry and ultrasonography imaging. All the patients underwent cholecystectomy surgery and randomized into two groups. Ten patients were treated with DNT, and the other ten patients received normal antibiotics. To determine the impact of DNT on bile acid homeostasis, the concentrations of serum bile acids including GCDCA, GCA and CA were analyzed by liquid chromatograph-mass spectrometer (LC-MS) (Fig. 1A). We found that serum levels of GCDCA, GCA and CA were abnormally higher in cholecystitis patients compared to the healthy people. Cholecystectomy operation followed by DNT therapy significantly reduced these bile acids compared to surgery followed by normal antibiotics. We also determined the serum inflammatory cytokine signatures using protein array (Fig. 1B, Supplementary Table S1). Intriguingly, PCA analysis clearly distinguished the four groups, and patients treated with DNT are more closely related to the healthy subjects (Fig. 1B). Our data indicated that DNT supplement following cholecystectomy operation alleviated the cholestasis symptoms and inflammatory responses of the cholecystitis patients.

Similar findings were recapitulated in a rodent cholestasis model induced by ANIT compound. Our data revealed that elevated serum levels of various bile acids, including TCA, THDCA, TCDCA and TUDCA, were significantly reduced by DNT treatment in ANIT-induced cholestatic mice (Fig. 1C). In addition, we examined the expression levels of inflammation-related genes. DNT treatment resulted in the downregulation of inflammatory genes evoked by ANIT exposure including IL-4, IL-6, IL-10, IL-1β, TGF-β1/2/3 and TNF-α (Supplementary Table S2). The PCA plot based on inflammatory gene expressions demonstrated that DNT treated mice formed a distinct cluster and more correlated with control group (Fig. 1D). Taken together, DNT reduced pathological bile acid accumulation and inflammation in cholecystitis patients and rodents.

Since imbalanced bile acid homeostasis and sustained inflammatory responses are both associated with cholestasis^12^,^13^, we further investigated the potential interaction between bile acids and inflammation. To address this point, mouse primary hepatocytes were treated with reconstituted bile acids (BA) milieu composed of TCA, βMCA, TαMCA and TβMCA, which is similar composition in the serum of ANIT treated mice. The bile acids pool did not show significant difference in cell viability (Fig. 1E). However, it substantially stimulated inflammatory responses, as indicated by the dramatic induction of *IL-2*, *IL-6*, *IL-10*, *IL-1β*, *iNOS*, *iCAM1* and *TNFα* gene expressions (Fig. 1F). Additionally, it is noteworthy that the markers for fibrosis, including *αSMA, Co1A1, MMP13* and *TGF1β* were remarkably increased upon bile acid exposure (Fig. 1G), suggesting that bile acid overload in hepatocytes had a profound effect on the development of fibrosis. These data imply that bile acids may, at least partially, contribute to unfavorable chronic inflammation often observed during cholestasis.

### DNT and curcumin activated FXR and mitigated cholestasis

**Figure 2:**
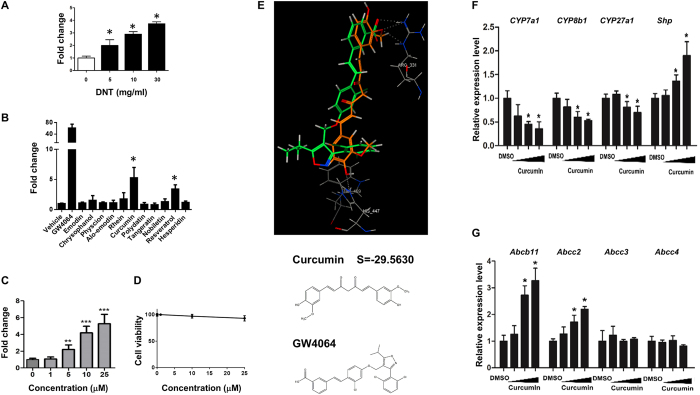
DNT and curcumin activated FXR *in vitro.* (**A**) HEK293T cells were exposure to dose-dependent concertation of DNT for 24 h after transfected with FXR plasmid DNA for 6 h. (**B**) Emodin, chrysophanol, physcion, alo-emodin, rhein, curcumin, polydatin, tangeratin, nobiletin, resveratrol and hesperidin, main compounds extracted from DNT, were studied for FXR activation. The doses of all compounds were used at 20 μM, while GW4064, a FXR synthesized agonist, at dose of 10 μM was used as positive control. (**C,D**) Curcumin at dose of 1 μM, 5 μM, 10 μM and 25 μM were treated in HEK293T cells after promoter region of FXR downstream gene and FXR overexpression plasmid co-transfection. FXR activity (**C**) and cell viability (**D**) were measured by the Dual-luciferase Reporter system and CCK-8 kit, respectively. (**E**) Molecular docking analysis between curcumin and FXR by computer simulation. GW4064 served as FXR docking template. Curcumin docking value with FXR was -29.5630 and potentially considered as FXR agonist. (**F,G**) Huh7 cells were treated with DMSO or curcumin (20 μM) for 24 h. Target genes regulated by FXR were studied by Real-time PCR. Bile acid synthesis and efflux genes, including *Cyp7a1*, *Cyp8b1*, *Cyp27a1*, *Shp*, *Abcb11*, *Abcc2*, *Abcc3* and *Abcc4* were involved. Data are presented as mean ± SD. *p < 0.05.

Since FXR plays a vital role in regulating bile acids and inflammation pathway^14^,^15^, we tested whether DNT exerted protective effects against cholestasis through modulating FXR activities. Interestingly, DNT exposure resulted in a dose-dependent activation of FXR (Fig. 2A), as indicated by the FXR reporter assay. To elucidate the candidate functional ingredient from DNT, herbal compounds were isolated and screened using FXR reporter assay (Fig. 2B). Two compounds, curcumin and resveratrol, induced FXR activity. As curcumin exhibited the most prominent impact on FXR activity, we focus on curcumin and continued the further study. The results showed FXR activity increased at a dose-dependent manner without cell toxicity (Fig. 2C,D). Molecular docking experiment showed that curcumin could theoretically bind with FXR protein at the same site as GW4064 (Arr331, His447), which is a synthesized FXR agonist, with an energy score of −29.5630. Consistently, we found that the majority of FXR target genes were regulated by curcumin (Fig. 2F,G). These data collectively suggested that curcumin was the main FXR activator in the ingredient of DNT *in vitro*.

**Figure 3:**
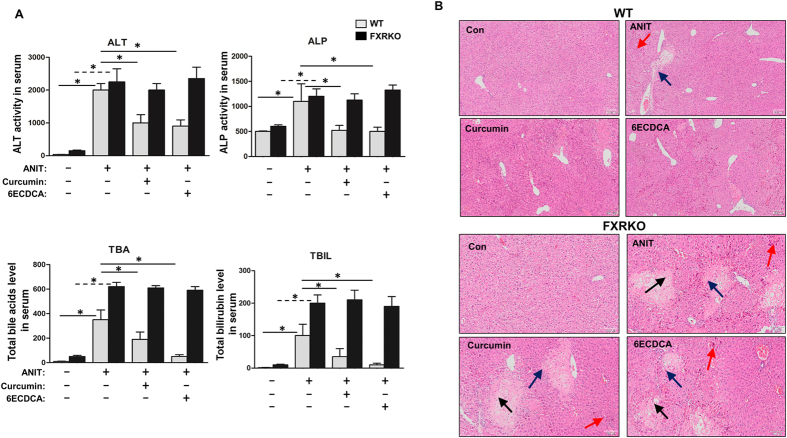
Curcumin protected ANIT-induced liver injury with cholestasis in presence of FXR by biochemical and histological analysis. (**A**) Serum TBA, ALP, ALT, DBIL and TBIL were detected in both WT and FXRKO mice serum. (**B**) Liver tissues from all groups in both WT and FXRKO mice were fixed and followed by H&E staining (black arrow: liver injury with ballooning; red arrow: cholestaisis; blue arrow: inflammation). Data are presented as mean ± SD. *p < 0.05.

Since curcumin activated FXR *in vitro*, we further investigated whether curcumin mitigated cholestasis symptoms through FXR *in vivo*. To this end, we evaluated the protective effect of curcumin against ANIT by biochemical and histological studies. Although the serum levels of biochemical markers of cholestasis, including ALT, ALP, TBA, DBIL and TBIL, were profoundly elevated after ANIT administration in WT mice, curcumin or 6ECDCA, which is a FXR agonist, improved the elevation of these markers (Fig. 3A). Surprisingly, neither curcumin nor 6ECDCA showed the inhibitory effect of the increased biochemical markers of cholestasis in FXRKO mice.

Histologically, ANIT-induced liver injuries, including inflammatory infiltration and parenchymal necrosis, were attenuated by curcumin or 6ECDCA pretreatment in WT mice (Fig. 3B upper and Supplementary Table S3). FXRKO mice demonstrated more obvious liver injuries in comparison to WT mice after ANIT administration, whereas curcumin or 6ECDCA did not ameliorate the injuries (Fig. 3B lower and Supplementary Table S3). Taken together, these observations implicated that curcumin ameliorated ANIT-induced cholestasis through FXR activation.

### Curcumin reduced hepatic bile acids accumulation after ANIT exposure in the presence of FXR

**Figure 4:**
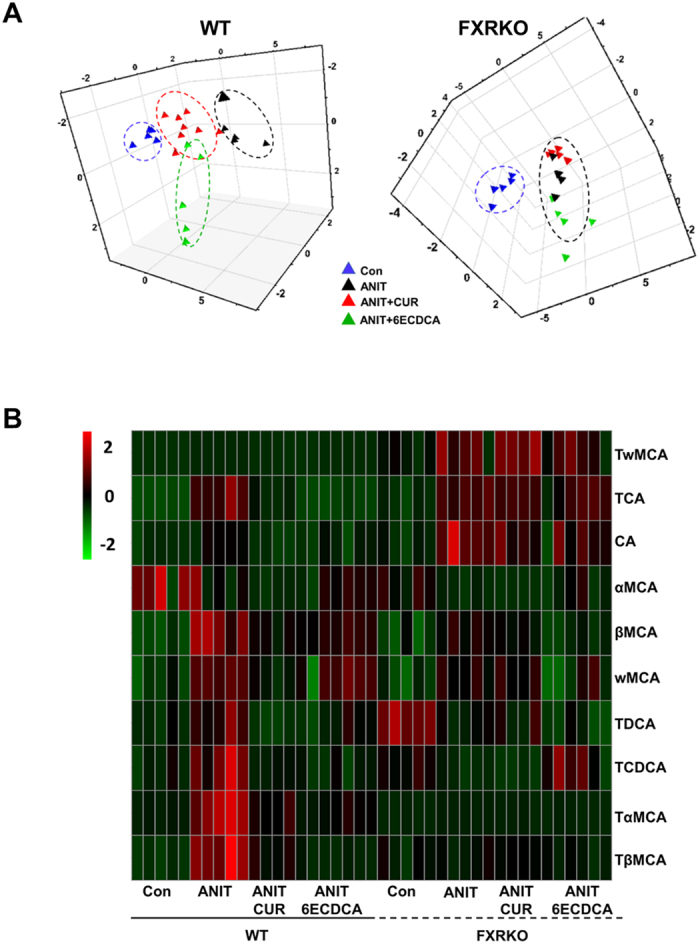
The effect of curcumin on ANIT-induced cholestasis through FXR-regulated bile acids profiles. Ten individual bile acids were quantified by LC-MS in both WT and FXRKO mouse livers, which were visualized in score plot of principal component analysis (PCA) (**A**) and heatmap (**B**). The colors on the heatmap correspond to the contents of bile acids Red represents the increase, while green represents the decrease. Hierarchical clustering separates X axis of heatmap represents different groups within 5 samples, while Y axis stands for bile acid levels.

Subsequently, we quantitated profiling of bile acids in a cholestasis mice model by LC-MS (Supplementary Figure S1). In WT mice, PCA analysis directly distinguishes the four groups. Either the ANIT+curcumin group or the ANIT+6ECDCA group is more closely related to the control group (Fig. 4A, left). Surprisingly, in FXRKO mice, the control group distributes furthest from the ANIT group, while the ANIT+curcumin and the ANIT+6ECDCA groups are scattered in a similar pattern to the ANIT group without noticeable discrepancy (Fig. 4A, right). These findings suggested that curcumin and 6ECDCA re-corrected the imbalance of bile acid homeostasis caused by ANIT in the presence of FXR. Furthermore, each hepatic bile acid was visualized in heatmap (Fig. 4B). In both WT and FXRKO mice, hepatic levels of CA, TCA, βMCA, ωMCA, TαMCA, TβMCA and TωMCA increased significantly after ANIT administration. As expected, these bile acids were dramatically recovered with curcumin or 6ECDCA treatment in ANIT-WT mice but not in ANIT-FXRKO mice. As a further proof, our data showed that curcumin protected from ANIT-induced cholestasis through reducing FXR-regulated bile acid overload in the liver.

### Curcumin normalized the imbalance of bile acid homeostasis caused by ANIT through FXR-regulated bile acid pathway

**Figure 5:**
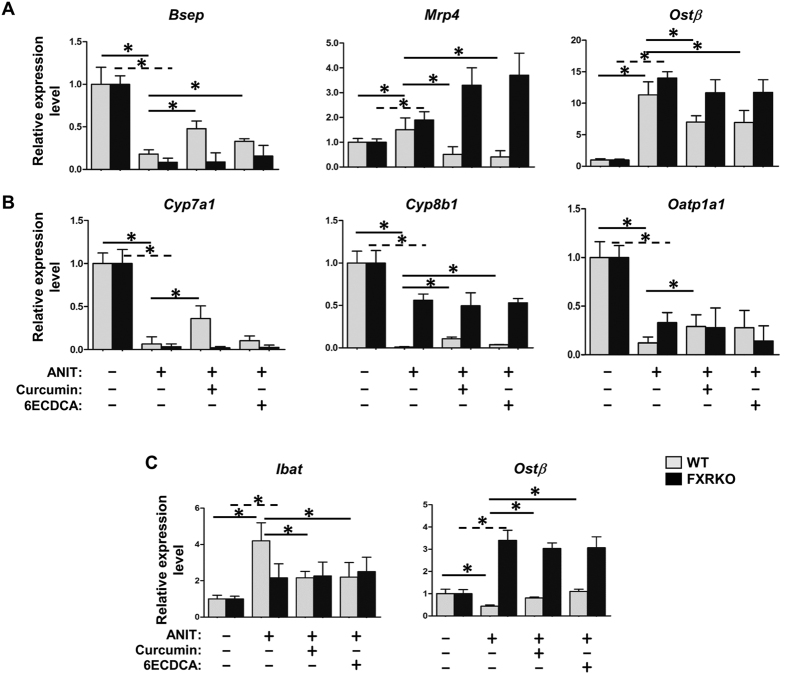
The effect of curcumin on bile acid signaling related genes after ANIT administration in WT and FXRKO mice. Liver and ileum tissues from both mice were used for gene expression. Bile acid transport (**A**), biosynthesis and uptake (**B**) and enterohepatic circulation related genes (**C**), including *Bsep*, *Mrp4*, *Ostβ*, *Cyp7a1*, *Cyp8b1*, *Oatp1a1* and *Ibat* were detected by Real-time PCR. Data are presented as mean ± SD. *p < 0.05.

To deeply examine the protective effect of curcumin against ANIT-induced cholestasis, some representative genes related to the bile acid signaling pathway were detected. First of all, we focused on the expressions of bile acid excretory transporters, including *Bsep*, *Mrp4* and *Ostβ* (Fig. 5A). ANIT treatment resulted in pronounced repression of *Bsep*, and induction of *Mrp4* and *Ostβ* expressions in WT mice (Fig. 5A). With curcumin or 6ECDCA treatment, the expression levels of these genes were correspondingly restored. Although these genes showed similar expression patterns in ANIT-treated FXRKO mice, neither curcumin nor 6ECDCA showed the rescue. Secondly, the genes in bile acid synthesis and uptake pathway, including *Cyp7a1*, *Cyp8b1* and *Oatp1a1*, were examined (Fig. 5B). ANIT treatment significantly downregulated these gene expression levels in WT mice, and curcumin or 6ECDCA treatment canceled these downregulation. While ANIT treatment suppressed these gene expressions in FXRKO mice, curcumin and 6ECDCA were unable to restore them. Finally, considering the importance of bile acid enterohepatic circulation on liver injury restoration, we therefore investigated the expressions of *Ibat* and *Ostβ* in ileums of all groups (Fig. 5C). ANIT treatment resulted in marked induction of *Ibat* expression as well as repression of *Ostβ* in the ileums of both types of mice. Similar as Fig. 5A,B, curcumin and 6ECDCA treatment restored these expressions in WT mice, not in FXRKO mice. These data highlighted the dominate impact of curcumin on bile acid signaling pathway through FXR regulation, resulting in inhibition of bile acid synthesis, acceleration of the bile acid efflux system and the normalization of the bile acid pool.

### Curcumin reduced inflammatory responses caused by ANIT in the presence of FXR

**Figure 6:**
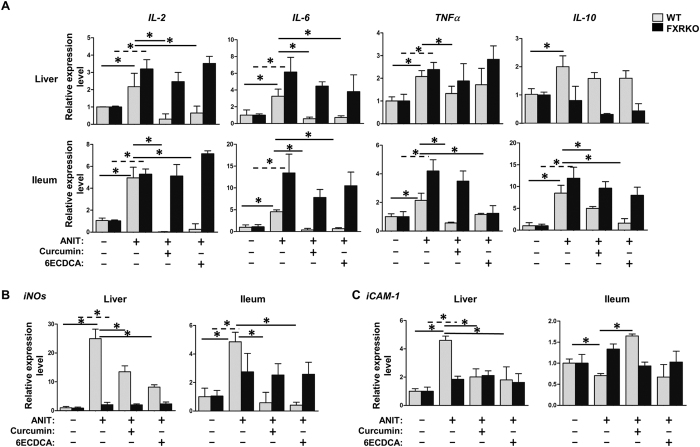
The effect of curcumin on inflammatory signaling related genes after ANIT administration in WT and FXRKO mice. Liver and ileum tissues from both WT and FXRKO mice were measured gene expression. Inflammation related genes, including *IL-2*, *IL-6*, *IL-10*, *TNFα* (**A**), *iNOS*(**B**) and *iCAM-1*(**C**), were detected by Real-time PCR. Data are presented as mean ± SD. *p < 0.05.

Since imbalanced bile acid homeostasis and sustained inflammatory responses are both associated with cholestasis, both of which are driven by FXR, thereby we investigated inflammatory signaling genes in both liver and ileum tissues. Inflammatory cytokines including *IL-2*, *IL-6*, *IL-10* and *TNFα* were over-expressed in both liver and ileum of ANIT-WT or -FXRKO mice (Fig. 6A). Except for *IL-10*, curcumin or 6ECDCA treatment reduced the induction of these genes in ANIT-WT, not in ANIT-FXRKO mice. Similarly, *iNOS* and *ICAM-1* induced by ANIT were restored by curcumin or 6ECDCA treatment in ANIT-WT mice, but not in ANIT-FXRKO mice. All the results suggested that curcumin alleviated liver injury by reducing the FXR-regulated inflammatory responses.

## Discussion

Our data highlighted that DNT exerted its beneficial effect in both clinical and animal studies. Mechanistic insights first revealed that curcumin, as important ingredient of DNT, had a profound role in cholestasis treatment, and the underlying basis was through signaling pathway of FXR-regulated bile acid and inflammation.

Cholecystitis is commonly considered as a complication of gallstone diseases and highly companied with cholestasis symptoms. Nowadays, laparoscopic cholecystectomy represents the gold standard treatment of acute cholecystitis to avoid recurrent symptoms and the operation should be performed as early as possible^16^. Normally, the surgery accompanied with a high possibility of infection and operative complications, which affected the recovery of cholecystitis. Up to date, except for antibiotics, there are no effective medicines for the conservative therapy or the complementary therapy for improving symptoms after surgery. Hence, we studied the wildly used formula for cholecystitis in China and discovered DNT as the most effective one. Notably, we employed the translational medicine strategy by shifting from the clinical investigations to understand the molecular basis in rodent study. Our data pointed out that the beneficial effect of DNT performance was highly related to FXR regulation.

FXR emerged as a therapeutic target in treating gallbladder diseases (such as cholestasis) has become increasingly evident^17^. GW4064, and OCA, which are synthesized FXR agonists showed notable FXR activation and protected cholestasis in animal models^9^,^18^. In the present study, we screened the most prominent and highly concentrated compounds derived from DNT such as, emodin, chrysophanol, physcion, alo-emodin, rhein, curcumin, polydatin, tangeratin, nobiletin, resveratrol, and hesperidin. It was apparent that curcumin was the most promising compound that could trigger FXR, and the effect was further confirmed by different dose experiments and FXR downstream target expressions. Except of curcumin, resveratrol exhibited the modest FXR-activated effect as well, and published evidences showed the protective effect of resveratrol on cholestasis and inflammation was related to the regulation of bile acid metabolism^19^,^20^, implying the underlying mechanism was relied on FXR activation, which indeed warrants further investigation.

Current study showed that curcumin attenuated ethanol-induced hepatic steatosis through modulating Nrf2/FXR signaling in hepatocytes^21^. Although they used alcoholic model disease model, their findings support our data. In addition, accumulative evidences regarding curcumin-activated PPARγ have been reported^22^,^23^, and some of them showed curcumin exerted anti-inflammatory and anti-fibrotic effect through PPARγ activation^24^, However, Narala VR *et al*. showed curcumin does not bind to PPARγ and not induced its activation directly^25^, suggesting FXR, not PPARγ may mainly contributes to the above effects because these two molecules have been reported the deep crosstalk. We proved that the activation of FXR by curcumin *in vitro* study, and exhibited its anti-cholestatic effect *in vivo* by using FXRKO mice, thus verified the beneficial effect of curcumin dependent on FXR. Importantly, for the first time, we showed the FXR was the predominate mediator in curcumin attenuating cholestasis study.

Bile acids are mainly regulated by FXR, and are the major metabolites of cholesterol. Not only do they facilitate in the absorption of hydrophobic materials and the metabolism of lipids and carbohydrates, they also play a critical role in liver injury and cholestasis protection^26^. Bile acid homeostasis depends on an intact enterohepatic circulation, which is disrupted by cholestasis. Abnormal increases in the bile acid pool due to cholestasis could in turn damage hepatocytes^27^. There are very few evidences regarding the direct effect of curcumin on bile acids. In our study, FXR deficiency abrogated the protective effect of curcumin on cholestasis, where hepatic bile acid overload persisted. A substantial number of bile acids in the liver increased a hundred times after ANIT treatment, and with FXR presence, curcumin was able to decrease bile acid levels back to normal. This comprehensive effect was helpful to avoid the second hit of accumulative bile acids on hepatocytes.

On the other hand, FXR also controls the inflammation pathway^14^. Cholestatic liver injury can further lead to severely chronic liver diseases if pro-inflammatory and pro-fibrogenic cytokines production are not controlled well. Beyond its role in bile acid regulation, curcumin is well known to exert to a potent anti-inflammatory activity^28^,^29^,^30^,^31^. The above results identified curcumin protecting liver injury with cholestasis through FXR-regulated inflammatory signaling pathway. Our results were consistent with many previous studies that FXR inhibited the inflammation mediator genes, such as *TNFα*, *IL-1β*, *IL-6*, and *iNOS*^32^,^33^. Moreover, we found that curcumin had a greater impact on the inflammatory genes expressed in the ileum than in the liver. One plausible explanation is that curcumin is poorly absorbed following oral administration, thus the majority of the ingested curcumin is exposed to intestinal tract, where it can exert its anti-inflammatory effect. This interpretation, however, required for the further investigation.

The above findings raised the question of how interactions between bile acid and inflammatory cytokines, and whether overload hepatic bile acids can directly induce the hepatocytes necrosis and trigger the inflammatory responses. To address this point, bile acids exposure in primary mouse hepatocyte were conducted and resulted in a slight hepatocyte toxicity. In contrast to histological observations, necrosis was observed in a large area after ANIT treatment (Fig. 3B) whereas there were only a small portion of hepatocyte deaths *in vitro* (Fig. 1E), which may lead to the conclusion that abnormal bile acids accumulation was not the primary cause of liver injury. If so, bile acid release should be considered as an outcome of liver injury triggered by endogenous stimulus. Our results were consistent with other observations by using single bile acid or different bile acid composition exposure to cells, such as GCDCA, TCA, CDCA, DCA, CA and UDCA^34^,^35^,^36^,^37^. Consistent reports showed these bile acids did not cause apoptosis or necrosis but induced the inflammatory genes or affected the rate of DNA repair or proliferation^37^,^38^. Another report challenged the cell death by using bile acids in rat hepatocytes, LCA and CDCA at dose of 100 μM caused significant decrease of cell viability, while GCDCA, TCDCA and TUDCA, at more than 250 μM showed cell death^39^,^40^. Importantly, the above literatures illustrated the combination of individual bile acids would enhance the cell death. In these cases, whether bile acid directly induced cell apoptosis or necrosis still remained debated, especially dependent on the concentrations and different kinds of bile acids used in each studies. Our data parallels the recent findings revealing that overload of bile acids is proposed as a mechanism for the inflammatory responses in the cholestasis mouse model^12^,^36^, and the constant inflammation responses are the important factors to induce HSC activation, pro-fibrotic responses, and eventually fibrosis^41^. In present study, we observed the fibrosis-related genes were markedly induced by bile acid mixture exposure, indicating bile acids being as the important signals or stimulus to induce inflammation and fibrosis. Similar as ANIT-induced cholestasis model, bile duct ligation can develop hepatic fibrosis by 2–3 weeks, accompanied by marked increase in various pro-fibrotic mediators and elevation of bile acids^42^,^43^. However, we reasoned that the inflammatory responses were not only due to bile acid overload in the liver but also ANIT itself. Bile acids and inflammation-signaling pathway are complementary in ANIT-induced cholestasis, and allowed curcumin to exert its protective effect through FXR activation. Taken together, the strategies for removal of the injury stimulus, the reduction of inflammatory burden and hepatic fibrosis should be focused on the regulation of bile acid homeostasis.

Besides, in order to discover other signaling pathways which contributed to cholestasis, we did the microarray analysis in different treatment groups. Initially, a wide spectrum of genes were obtained by comparing the ANIT group and Con group in WT mice, and then the selected genes were performed a functional annotation by KEGG and GO pathway analysis. As shown in Supplementary Tables S4 and S5, bile secretion, steroid hormone biosynthesis, acute inflammatory response, were the important pathways which had a profound impact on the cholestasis and were thoroughly investigated in the present study. Besides, such as metabolism of xenobiotics by cytochrome P450, retinol metabolism, PI3K-Akt signaling and p53 signaling pathway were also responsible for the cholestasis, which provided a comprehensive picture of the function of cholestasis and still warrants further investigation. By using these genes, we observed the protective effect of curcumin against ANIT in WT mice by cluster analysis (Supplementary Figure S2).

In conclusion, our data highlighted the dominate role of DNT in benefiting the outcome of cholecystits in clinic and cholestasis in rodents. Several complementary lines of evidences supported that curcumin was the most important ingredient of DNT for cholestasis treatment, and offered an alternative treatment approach particularly in FXR-regulated bile acid and inflammation pathway.

## Materials and Methods

### Chemicals and reagents

Alpha-naphthylisothiocyanate (ANIT) and curcumin (CUR) were obtained from Sigma-Aldrich (Sigma-Aldrich, USA). All the bile acid standards were purchased from Sigma-Aldrich as well. Danning tablet used in this study were produced by Shanghai Huchison Pharmaceuticals Co. Ltd (batch No. Z10910040). Ammonium acetate, formic acid, acetonitrile and methanol (HPLC grade) were purchased from Fisher Scientific (Nepean, Ont, Canada). Ultrapure water was prepared by a Milli-Q50 SP Reagent Water System (Millipore Corporation, MA, USA) for the preparation of samples and buffer solutions.

### Clinical specimens

Plasma samples were collected from patients in Shuguang Hospital Affiliated with Shanghai University of Traditional Chinese Medicine. The symptom of the patients like unremitting right upper quadrant pain, anorexia, nausea, vomiting or fever lasted for several weeks. Ultrasonography imaging indicated thickening and rough gallbladder wall, distortional gallbladder with gallstones. They have surgical indication of laparoscopic cholecystectomy, open cholecystectomy or minilaparoscopic surgery. A detailed medical questionnaire was completed to ensure that subjects were eligible for the study and 20 patients participated in this study in the end. They are double-blind randomized into two groups after cholecystectomy operation. The treatment groups received Danning tablet regimen (15 tablets per day) for 12 weeks since 5 days after operation relative to the normal antibiotics therapy which control groups received. Their blood were collected at 13 weeks after the operation. All samples were prepared for detection of inflammatory cytokines and bile acid concentrations. The methods were carried out in accordance with the Ethical Guidelines for Medical and Health Research Involving Human Subjects. All subjects provided written informed consent and the study was approved by Institutional Ethical Committee of the Shuguang Hospital Affiliated with Shanghai University of Traditional Chinese Medicine (Approval Notice No. 2011LC3Y033).

### Cell culture

All the reagents used for cell culture were obtained from Gibco-BRL (Carlsbad, CA, USA) unless otherwise noted. Huh7 (Japanese Collection of Research Bioresources Cell Bank) and HEK293T cell lines (American Type Culture Collection) were cultured in Dulbecco’s modified Eagle’s medium supplemented with 10% fetal bovine serum. Primary mouse hepatocyte cells (Research Institute for Liver Disease (Shanghai)) was cultured in RPMI 1640 medium supplemented with 10% fetal bovine serum.

### Luciferase assay

HEK293T cells were transfected with βRE (FXR downstream gene promoter region), expressed FXR and RXRα plasmids for 6 h using Lipofectamine 2000 (Invitrogen, Carlsbad, CA). Then, the medium was replenished with fresh medium containing Danning tablet, compounds derived from DNT for 24 h. After treatment, cells were collected to measure firefly and renilla luciferase activity using the Dual-luciferase Reporter system (Promega, Madison, WI). Renilla luciferase activity was standardized to the Firefly luciferase activities.

### Quantification of mRNA

Total RNA was extracted using TRIzol reagent (Invitrogen, Carlsbad, CA), and reverse transcribed into cDNA with a High Capacity RNA-to-cDNA Kit (Applied Biosystems, CA), according to manufacturer instructions. The mRNA levels were quantified by real-time PCR on an ABI ViiA 7 Real time PCR system (Applied Biosystems, CA, USA) using Power SYBR Green PCR Master Mix (Applied Biosystems, CA, USA). *Gapdh* were used as internal controls to normalize the detected mRNAs. Primers were designed using Primer3 Input software and the primers sequences are available upon request.

### Animals

Wild type C57/BL mice were purchased from the Laboratory Animal Center of Shanghai University of Traditional Chinese Medicine (SHUTCM, Shanghai). FXRKO mice were transferred from UC Davis medical center, and reproduced in SHUTCM animal room. The mice were housed at 20 (±2)°C with relative humidity at 60–70%. The animal welfare strictly complied with the Guide for the Care and Use of Laboratory Animals, and the protocols for the animal experiments were approved by the Institutional Animal Committee of Shanghai University of Traditional Chinese Medicine (Permit number: SCXK (Hu) 2012-0002). Wild-type and FXRKO mice were randomly assigned into four groups (Control, ANIT, ANIT+curcumin, ANIT+6ECDCA), respectively. ANIT+curcumin and ANIT+6ECDCA were treated with curcumin (120 mg/kg) or 6ECDCA (20 mg/kg), respectively, for 5 days while Control and ANIT were given normal saline. (Curcumin at dose of 120 mg/kg or 6ECDCA at dose of 20 mg/kg was selected based on our previous study and published findings^44^,^45^,^46^). Four hours after curcumin or 6ECDCA treatment on day 2, ANIT (dissolved in oil, 60 mg/kg) were administered intragastrically in the ANIT, ANIT+curcumin, and ANIT+6ECDCA groups. In contrast, the control group were administered olive oil intragastrically. After 48 h ANIT administration, mice were anesthetized with isoflurane and euthanized. Another sets of WT mice were assigned into Control, ANIT and ANIT+DNT (Danning tablet) groups. The treatment were the same as described above. Blood samples were coagulated in 1.5 h followed by centrifugation to obtain serum at 4 °C (3000× g). Livers and ileums were frozen in liquid nitrogen immediately after collection and stored in −80 °C freezer for further assays.

### Biochemical assay and histological study

Alkaline phosphatase (ALP), total bilirubin (TBIL), aspartate aminotransferase (ALT) and the concentration of total bile acid (TBA) were measured using an automated biochemistry analyzer (Olympus 2700, Japan). Livers were stained with a standard hematoxylin and eosin (H&E) procedure to reveal patterns of cellular labeling under the light-microscope for necrosis and other structural changes. Serum inflammatory cytokines (CD14, CD27, CD30, CD40, CD40L, CRP, CXCL16, IFNγ, IL-10, IL-18 Rb, IL-1α, IL-4, IL-6, IL-8, MCP-1, TGFβ RIII, TGF-β1, TGF-β2, TGF-β3 and TNFα) were detected by Wayen Biotechnology Company using Human Antibody Array (QAH-CYT-SW).

### Quantification of bile acids in mouse livers

An ultra-performance liquid chromatography instrument coupled with a quatropole mass spectrometry (UPLC-MS, Waters Co., MA, USA) was used to detect hepatic bile acids. Livers were homogenized in acetonitrile (100 mg tissue/500 μl acetonitrile) followed by centrifugation at 14,300 rpm for 10 min. The supernatant was blown to dryness under nitrogen stream, then re-dissolved in methanol-water solution (methanol:water:formic acid = 50:50:0.01) followed by centrifugation at 14,300 rpm for 10 min. Samples of 5 μl of the supernatants were injected into the UPLC-MS instrument. The instrument parameters were applied based on previous study^47^.

### Molecular docking

To evaluate the activity of curcumin, the docking program Molegro Virtual Docker (MVD) was used to dock the structure of FXR (PDB code: 3DCT, FXR with GW4064). Before docking analysis, original structure of all crystal water molecules should be removed and added hydrogen in the DS CDOCKER module. The compound should start with the lowest energy state to reach an optimal starting conformation before docking. Based on reported docking sites in GW4064 docking analysis, such as Arg331, Trp469, and His447 were bond with GW4064 by hydrogen, thereby the conformation of curcumin was evaluated based on those bond status. In the end, there is an energy score output to evaluate curcumin being as FXR agonist or not.

### *In vitro* study

Mouse primary hepatocytes derived from male C57/BL mice were purchased from Shanghai Research Institute Liver Disease Co. Ltd (originally from Celsis *in vitro* technologies). Mouse primary hepatocytes was cultured with DMEM medium supplemented with 10% fetal bovine serum. Cells were treated with reconstituted BA milieu, composition of which comes from the high concentration and most elevated individual bile acids after ANIT administration in *in vivo* study. (TCA 10 μg/ml, TDCA 18 μg/ml, βMCA 18 μg/ml, TαMCA 18 μg/ml and TβMCA 120 μg/ml). Cell viability was studied using CCK-8 assay kit according to manufacturer instructions (Shanghai Yeasen Biotech, Shanghai, China). Inflammation-related and fibrosis-related gene expressions were detected by Real-time PCR.

### Data analysis

One-way ANOVA statistic and PCA algorithm were performed by SPSS 18.0 (SPSS Inc. Chicago, IL, USA). PCA plot was performed by SIMCAP (11.5 version, Umetrics, Ume, Sweden) and heatmap was imaged by The R Programming Language. All graphs were generated with the Graphpad software (GraphPad Inc, San Diego, CA).

## Additional Information

**How to cite this article**: Yang, F. *et al*. Curcumin protects ANIT-induced cholestasis through signaling pathway of FXR-regulated bile acid and inflammation. *Sci. Rep.* **6**, 33052; doi: 10.1038/srep33052 (2016).

## Supplementary Material

Supplementary Information
