# Supplementary material for: Curcumin protects ANIT-induced cholestasis through signaling pathway of FXR-regulated bile acid and inflammation
Source: Sci Rep. 2016 Sep 14;6:33052. doi: 10.1038/srep33052 (PMC5021964; doi:10.1038/srep33052)
Supplement: Supplementary Information [file srep33052-s1.doc]

**Supplementary information:**

**Curcumin protects ANIT-induced cholestasis through signaling pathway of**

**FXR-regulated bile acid and inflammation**

**(SREP-16-14210A)**

**Fan Yang^1+^, Xiaowen Tang^1+^, Lili Ding^1^, Yue zhou^1^, Qiaoling Yang^1^, Junting Gong^1^, Guangyun Wang^1^, Zhengtao Wang^1*^, Li Yang^1, 2*^**

^1^ The MOE Key Laboratory for Standardization of Chinese Medicines and the SHTCM Key Laboratory for New Resources and Quality Evaluation of Chinese Medicines, Institute of Chinese Materia Medica, Shanghai University of Traditional Chinese Medicine, Shanghai 201203, China

^2^ Center for Chinese Medical Therapy and Systems Biology, Shanghai University of Traditional Chinese Medicine, Shanghai 201203, China

^+^ Contributed equally to this manuscript.

* Correspondence authors:

Addresses: Institute of Traditional Chinese Materia Medica, Shanghai University of Traditional Chinese Medicine, 1200 Cailun Road, Shanghai 201210, China.

Tel: +8651322507; fax: +862151322519.

E-mail: Zhengtao Wang (ztwang@shutcm.edu.cn); Li Yang ([yangli7951@hotmail.com](mailto:yangli7951@hotmail.com); [yl7@shutcm.edu.cn](mailto:yl7@shutcm.edu.cn))

*Supplementary table S1. The expression of each inflammatory cytokines in patients’ serum (related to Figure 1B).*

|  | **pre** | **health** | **control** | **treatment** |
| --- | --- | --- | --- | --- |
| CD14 | 1833.41±123.26 | 1150.05±162.74 | 833.07±211.35 | 1090.13±464.75 |
| CD27 | 172.76±32.68 | 25.18±9.75 | 92.60±19.53 | 83.57±23.24 |
| CD30 | 126.19±28.20 | 44.44±5.09 | 53.94±9.83 | 61.81±4.33 |
| CD40 | 127.53±37.40 | 43.61±4.09 | 82.15±43.39 | 66.78±20.92 |
| CD40 L | 771.29±101.51 | 338.36±39.45 | 519.18±122.62 | 186.67±8.90 |
| CRP | 2561.66±184.83 | 454.38±83.18 | 1751.42±432.75 | 1351.82±259.17 |
| CXCL16 | 3553.70±265.57 | 1392.06±94.47 | 1894.80±260.06 | 1776.16±411.76 |
| IFNg | 193.97±26.78 | 96.13±17.84 | 118.62±14.16 | 104.55±11.48 |
| IL-10 | 175.53±27.47 | 95.23±9.68 | 103.82±18.89 | 97.52±12.74 |
| IL-18 Rb | 193.00±29.61 | 103.64±3.70 | 164.93±25.68 | 185.63±59.72 |
| IL-1a | 194.95±33.69 | 137.05±22.97 | 270.77±24.52 | 177.94±29.00 |
| IL-4 | 74.50±22.55 | 29.26±8.33 | 31.84±9.57 | 37.77±19.35 |
| IL-6 | 149.67±27.63 | 113.93±25.74 | 79.00±10.49 | 73.05±15.39 |
| IL-8 | 178.79±28.16 | 99.90±10.24 | 112.49±15.43 | 99.62±11.47 |
| MCP-1 | 189.75±44.64 | 96.91±7.42 | 102.98±17.69 | 86.93±16.84 |
| TGFb RIII | 283.94±52.22 | 114.71±59.02 | 163.70±86.08 | 141.41±36.75 |
| TGF-b1 | 203.64±27.24 | 147.06±23.81 | 119.32±29.10 | 127.78±20.98 |
| TGF-b2 | 192.40±30.00 | 89.78±12.69 | 126.69±8.22 | 98.75±11.85 |
| TGF-b3 | 132.49±51.98 | 27.54±5.95 | 183.59±188.95 | 203.51±121.45 |
| TNFa | 179.89±27.55 | 86.52±10.82 | 121.63±20.61 | 101.03±20.45 |

Supplementary table S2. The expression of each inflammatory cytokines in rodent models’ serum (related to Figure 1D).

|  | **Con** | **ANIT** | **DNP** |
| --- | --- | --- | --- |
| IL-4 | 1±0.32 | 3.73±0.73 | 0.90±0.28 |
| IL-6 | 1±0.17 | 3.94±1.08 | 0.94±0.18 |
| IL-10 | 1±0.10 | 0.41±0.12 | 0.54±0.10 |
| IL-1b | 1±0.06 | 4.93±1.03 | 1.15±0.06 |
| TGF-B1 | 1±0.19 | 2.01±0.23 | 1.28±0.26 |
| TGF-b2 | 1±0.05 | 3.85±0.88 | 1.69±0.65 |
| TGF-b3 | 1±0.17 | 1.52±0.31 | 0.59±0.17 |
| TNFa | 1±0.35 | 2.7±0.70 | 0.90±0.26 |

Supplementary table S3. Liver damage index in different treatment groups (n=6).

|  |  |  |  |  | **Count (x/6)** |  |  |  |  |
| --- | --- | --- | --- | --- | --- | --- | --- | --- | --- |
| **Item** | **Definition** | **Con** | **ANIT** | **ANIT CUR** | **ANIT**  **6ECDCA** | **Con** | **ANIT** | **ANIT**  **CUR** | **ANIT**  **6ECDCA** |
|  |  |  |  | **WT** |  |  |  | **FXRKO** |  |
| **Inflammation** | No foci | 6/6 |  |  | 1/6 | 4/6 |  |  |  |
|  | <2 foci |  |  | 4/6 | 3/6 | 2/6 |  |  |  |
|  | 2-4 foci |  | 3/6 | 2/6 | 2/6 |  | 1/6 | 2/6 | 3/6 |
|  | >4 foci |  | 3/6 |  |  |  | 5/6 | 4/6 | 3/6 |
| **Liver injury** | None | 4/6 |  |  | 2/6 | 1/6 |  |  |  |
| **(ballooning)** | Few | 2/6 |  | 4/6 | 3/6 | 2/6 |  |  |  |
|  | Many |  | 6/6 | 2/6 | 1/6 | 3/6 | 6/6 | 6/6 | 6/6 |
| **Cholestasis** | None | 6/6 |  |  | 3/6 | 6/6 |  | 1/6 |  |
|  | Mild |  | 1/6 | 5/6 | 3/6 |  |  |  | 1/6 |
|  | Moderate |  | 5/6 | 1/6 |  |  | 6/6 | 5/6 | 5/6 |
| **Steatosis** | None | 6/6 |  | 6/6 | 6/6 | 6/6 |  |  |  |
|  | Few |  | 6/6 |  |  |  | 5/6 | 4/6 | 3/6 |
|  | Many |  |  |  |  |  | 1/6 | 2/6 | 3/6 |

Supplementary table S4. TOP25 pathways contributed to cholestasis (KEGG)

| **ID** | **Description** | **P value** | **Gene Symbol** |
| --- | --- | --- | --- |
| 00100 | Steroid biosynthesis | 0.000286 | *Cyp51/Fdft1/Lss/Nsdhl/Sqle/Sc5d/*  *Msmo1/Dhcr24* |
| 04060 | Cytokine-cytokine receptor interaction | 0.000579 | *Cxcr5/Cxcr2/Cxcr3/Ccr2/Ackr3/Csf3r/*  *Edar/Cxcl1/Cxcl10/Ifng/Il12a/Il12rb1/*  *Il1a/Il6/Il7r/Kdr/Lifr/Osmr/Ccl21a/Prlr/*  *Ccl20/Ccl3/Ccl4/Ccl7/Cxcl2/Cxcl5/Il23r/*  *Tnfrsf10b/Tnfrsf8/Tnfsf12/Tnfsf8/Tnfsf9/*  *Tnfsf10/Tnfsf18/Ifnlr1/Eda2r/Tnfrsf19/*  *Tnfsf15/Il19/Tslp/Cxcl13/Tnfrsf13c/Il23a*  */Tnfrsf25* |
| 04080 | Neuroactive ligand-receptor interaction | 0.001481 | *Grm2/Chrna5/Chrna2/Adcyap1r1/Adora3/*  *Adra2a/Agtr1b/Bdkrb2/C5ar1/Cckbr/*  *Chrm3/Drd5/S1pr4/Gabra3/Gabrb2/*  *Gabrg2/Galr2/Glp1r/Grid1/Grid2/Grik3/*  *Grin2b/Grm8/Hrh2/Htr2c/Lhcgr/Trhr2/*  *Mas1/Mtnr1a/Npy6r/Prlr/Ptger1/Ptger3/*  *Ptger4/Ptgfr/Pth2r/Thra/Chrna9/Grm4/*  *Gipr/Gria3/Calcrl/P2ry4/Gpr35/S1pr5* |
| 00900 | Terpenoid backbone biosynthesis | 0.001758 | *Fdps/Hmgcr/Hmgcs2/Mvk/Mvd/*  *Hmgcs1/Idi1/Nus1* |
| 00140 | Steroid hormone biosynthesis | 0.005557 | *Cyp1a1/Cyp1a2/Cyp1b1/Cyp2c38/*  *Cyp2c39/Cyp7a1/Hsd11b1/Hsd17b1/*  *Hsd3b4/Hsd3b5/Sult1e1/Cyp2c54/*  *Sult2b1/Cyp2c66/Ugt2b1/Ugt2a3/Ugt2a1* |
| 00830 | Retinol metabolism | 0.007032 | *Rdh9/Cyp1a1/Cyp1a2/Cyp2c38/*  *Cyp2c39/Cyp4a10/Cyp4a14/Dhrs9/*  *Cyp2c54/Cyp4a31/Cyp2c66/Aox3/*  *Ugt2b1/Ugt2a3/Cyp2s1/Lrat/Ugt2a1* |
| 00590 | Arachidonic acid metabolism | 0.00788 | *Cyp2j11/Alox5/Cbr2/Cyp2c38/Cyp2c39/*  *Cyp4a10/Cyp4a14/Pla2g5/Cyp2j13/*  *Cyp2c54/Hpgds/Cyp4f14/Plb1/*  *Cyp4a31/Cyp2c66/Cyp2u1/Cyp4f18* |
| 04512 | ECM-receptor interaction | 0.008029 | *Col4a3/Col4a4/Col6a1/Hmmr/Itga2/Itgb6/*  *Lama2/Lama4/Reln/Thbs1/Thbs2/Itga8/*  *Itga11/Sv2c/Tnxb/Col4a6* |
| 04976 | Bile secretion | 0.010425 | *Slc22a7/Aqp4/Aqp8/Cyp7a1/Hmgcr/*  *Abcb1b/Nr1h4/Slc10a2/Slc5a1/Slc4a5/*  *Nr0b2/Slco1a1/Slco1a6/Slc51b* |
| 00980 | Metabolism of xenobiotics by cytochrome P450 | 0.011844 | *Cbr2/Cyp1a1/Cyp1a2/Cyp1b1/Cyp2f2/*  *Gsta2/Hsd11b1/Mgst2/Hpgds/Ugt2b1/*  *Ugt2a3/Cyp2s1/Ugt2a1* |
| 04110 | Cell cycle | 0.013144 | *Cdc20/Ccnd1/Ccne1/Ccne2/Cdc25a/Cdc45/Cdkn1a/Cdkn1c/Chek1/Mcm3/Mcm4/*  *Mcm5/Gadd45b/Orc1/Plk1/Cdc6/Orc5/*  *Ccnb1/Pkmyt1/Wee2/Cdc26* |
| 04151 | PI3K-Akt signaling pathway | 0.016405 | *Bcl2l1/Brca1/Ccnd1/Ccne1/Ccne2/Cd19/*  *Cdkn1a/Col4a3/Col4a4/Col6a1/Csf3r/Fgf12/Fgf14/Fgf15/Fgf2/Fgf9/Fgfr2/Gnb3/*  *Hsp90aa1/Igf1/Il6/Il7r/Itga2/Itgb6/Kdr/*  *Lama2/Lama4/Sgk3/Myb/Osmr/Prkca/*  *Prlr/Reln/Creb3l2/Tek/Thbs1/Thbs2/Tlr2/*  *Itga8/Ppp2r2c/Tcl1b1/Itga11/Foxo3/*  *Rps6kb1/Pik3cb/Tnxb/Pik3ap1/Col4a6* |
| 00380 | Tryptophan metabolism | 0.016991 | *Cyp1a1/Cyp1a2/Cyp1b1/Tph2/Inmt/*  *Acmsd/Afmid/Aox3/Ehhadh/Hadha* |
| 00512 | Mucin type O-Glycan biosynthesis | 0.0186 | *Galnt7/Galnt3/St6galnac1/Galnt18/*  *B4galt5/Galnt14/Galnt15* |
| 05410 | Hypertrophic cardiomyopathy (HCM) | 0.019846 | *Prkag2/Ace/Actg1/Cacna1d/Igf1/Il6/*  *Itga2/Itgb6/Lama2/Lmna/Ryr2/Slc8a1/*  *Tnnc1/Itga8/Itga11* |
| 05144 | Malaria | 0.025624 | *Ackr1/Gypa/Ifng/Il12a/Il6/Thbs1/Thbs2/*  *Vcam1/Tlr2/Gypc* |
| 04020 | Calcium signaling pathway | 0.02604 | *Phkb/Agtr1b/Bdkrb2/Cacna1b/Cacna1d/*  *Camk4/Cckbr/Chrm3/Drd5/Erbb4/Hrh2/*  *Htr2c/Lhcgr/Trhr2/Prkca/Plcd4/Ptger1/*  *Ptger3/Ptgfr/Ptk2b/Ryr2/Slc8a1/Tnnc1/*  *Itpkc/Slc25a31/Plce1/Calm4* |
| 04750 | Inflammatory mediator regulation of TRP channels | 0.026983 | *Cyp2j11/Asic1/Bdkrb2/Cyp2c38/Cyp2c39/*  *Cyp4a10/Cyp4a14/Htr2c/Igf1/Asic3/*  *Prkca/Prkcq/Ptger4/Cyp2j13/Map2k6/*  *Cyp2c54/Cyp4a31/Cyp2c66/Pik3cb/*  *Calm4* |
| 04913 | Ovarian steroidogenesis | 0.028536 | *Cyp2j11/Alox5/Bmp6/Cyp1a1/Cyp1b1/*  *Hsd17b1/Hsd3b4/Hsd3b5/Igf1/Lhcgr/*  *Cyp2j13* |
| 04640 | Hematopoietic cell lineage | 0.029192 | *Cd19/Cd2/Cd4/Cd8a/Csf3r/Cd55/Fcer2a/*  *Il1a/Il6/Il7r/Itga2/Mme/Dntt/Tfrc/Cd59b* |
| 00670 | One carbon pool by folate | 0.0363 | *Mthfsl/Ftcd/Mthfd2/Mthfr/Aldh1l2* |
| 04115 | p53 signaling pathway | 0.037922 | *Ccnd1/Ccne1/Ccne2/Cdkn1a/Chek1/*  *Igf1/Igfbp3/Gadd45b/Serpine1/Thbs1/*  *Ccnb1/Pidd1* |
| 05323 | Rheumatoid arthritis | 0.044364 | *Ctla4/Fos/H2-Oa/Ifng/Il1a/Il6/Ccl20/Ccl3/*  *Cxcl5/Tek/Tlr2/Atp6v1g3/Il23a/Mmp1a* |
| 00650 | Butanoate metabolism | 0.04865 | *Hmgcs2/Hmgcs1/Acsm2/Oxct1/Ehhadh/*  *Hadha* |
| 04514 | Cell adhesion molecules (CAMs) | 0.049211 | *Alcam/Ctla4/Cd2/Cd4/Cd8a/Cdh15/Cdh4/*  *Cldn4/Vcan/H2-Oa/Mpz/Ncam2/Ptprm/Siglec1/*  *Sdc3/Cd226/Itga8/Lrrc4c/Negr1/Icosl/Cntnap1/Cldn8/Cldn23/Cldn34c4* |

Supplementary table S5. Top 25 pathways contributed to cholestasis (GO)

| **ID** | **Description** | **Enrich factor** | ***Gene Symbol*** |
| --- | --- | --- | --- |
| GO:0008299 | isoprenoid biosynthetic process | 5.19 | *Rdh9/Fdps/Cyp1a1/Fdft1/Hmgcr/Hmgcs2/Mvk/Mvd/Hmgcs1/Dhrs9/Idi1/Nus1* |
| GO:0070098 | chemokine-mediated signaling pathway | 4.46 | *Cxcr2/Cxcr3/Ackr3/Ackr1/Cxcl1/Cxcl10/Ptk2b/Cxcl2/Cxcl5/Slit3/Tff2/Cxcl13/Trem2* |
| GO:0016126 | sterol biosynthetic process | 3.65 | *Fdps/Apoa4/Cyp51/Fdft1/Hmgcr/Hmgcs2/Lss/Mvk/Nsdhl/Mvd/Hmgcs1/Sc5d/Npc1l1/Msmo1/Dhcr24* |
| GO:0046165 | alcohol biosynthetic process | 2.83 | *Fdps/Adcyap1r1/Apoa4/Bmp6/Cyp51/Fdft1/Fgf2/Gfi1/Hmgcr/Hmgcs2/Hsd17b1/Ifng/Fabp5/Lhcgr/Lss/Mas1/Mvk/Nsdhl/Pla2g5/Mvd/Ptk2b/Snai2/Sptlc2/Hmgcs1/Sc5d/Npc1l1/Dkk3/Nus1/Msmo1/Isyna1/Dhcr24* |
| GO:0006694 | steroid biosynthetic process | 2.81 | *Fdps/Akr1c20/Apoa4/Bmp6/Cyp51/Cyp7a1/Fdft1/Fgf15/Gfi1/Hmgcr/Hmgcs2/Hsd11b1/Hsd17b1/Hsd3b5/Ifng/Igf1/Il1a/Lhcgr/Lss/Mvk/Nsdhl/Mvd/Nr1h4/Snai2/Hmgcs1/Nr1d1/Pde8b/Sc5d/Npc1l1/Dkk3/Msmo1/Dhcr24/Akr1c21/Akr1c6* |
| GO:0019722 | calcium-mediated signaling | 2.75 | *Ank2/Adora3/Cacna1d/Casq2/Cd4/Cd8a/Cxcr3/Fcer1a/Ptgdr2/Igf1/Kdr/Lhcgr/Bhlha15/Ntrk2/Ptgfr/Ptk2b/Ryr2/Ccl4/Exoc4/Slc8a1/Tff2/Vcam1/Alms1/Ppp1r9a/Adgrl1/Rcan1/Cmya5/Trem2* |
| GO:0002526 | acute inflammatory response | 2.65 | *Adam8/Adra2a/Alox5/Bdkrb2/Cxcr2/Cr1l/Cd55/Ephb6/Fcer1a/Cxcl1/Il1a/Il1rn/Il6/Itih4/Klk1b1/Orm1/Orm2/Reg3b/Ptger3/Saa3/Saa4/Tac1/A2m/Ffar2/Cd59b* |
| GO:0050921 | positive regulation of chemotaxis | 2.64 | *C5ar1/Casr/Cxcr2/Cxcr3/Ccr2/Fgf2/Cxcl1/Hspb1/Cxcl10/Il12a/Itga2/Kdr/Nrp1/Prkca/Serpine1/Ccl21a/Ptk2b/Ccl4/Cxcl2/Cxcl5/Snai2/Stx3/Thbs1/Tnfsf18/Cxcl13/Il23a* |
| GO:0048520 | positive regulation of behavior | 2.50 | *C5ar1/Casr/Cxcr2/Cxcr3/Ccr2/Fgf2/Cxcl1/Hspb1/Cxcl10/Il12a/Itga2/Kdr/Nrp1/Prkca/Serpine1/Ccl21a/Ptger3/Ptger4/Ptk2b/Ccl4/Cxcl2/Cxcl5/Snai2/Stx3/Thbs1/Tnfsf18/Uts2/Cxcl13/Ghrl/Sgip1/Il23a* |
| GO:0019932 | second-messenger-mediated signaling | 2.42 | *Ank2/Adcyap1r1/Adora3/Rims2/Cacna1d/Casq2/Cd4/Cd8a/Cxcr3/Fcer1a/Glp1r/Ptgdr2/Htr2c/Cxcl10/Igf1/Kdr/Lhcgr/Bhlha15/Mrvi1/Mt1/Npr3/Ntrk2/Ptgfr/Ptk2b/Rasd1/Ryr2/Ccl4/Exoc4/Slc8a1/Tff2/Thbs1/Vcam1/Alms1/Ppp1r9a/Adgrl1/Rcan1/Rapgef4/Pex5l/Cmya5/Trem2* |
| GO:0005578 | proteinaceous extracellular matrix | 2.41 | *Spon2/Adamts9/Gpc5/Ltbp4/Alpl/Prelp/Col11a1/Col12a1/Col14a1/Col4a3/Col4a4/Col6a1/Col8a1/Hapln1/Vcan/Ncan/Epyc/Ecm1/Sparcl1/Col26a1/Fbln1/Fbn2/Fgf9/Igf1/Ihh/Lama2/Lama4/Lect1/Mmp2/Mmp24/Mmp7/Mmp8/Mmp9/Ogn/Reln/Serpinf1/Sparc/Thsd4/Efemp1/Adamts2/Thbs2/Timp3/Clec3b/Wnt10a/Wnt10b/Wnt2/Sbspon/Megf9/Spon1/Fbln5/Gpc6/Frem2/Otoa/Egflam/Ltbp1/Optc/Adamts16/Emilin3/Abi3bp/Hapln4/Postn/Hmcn1/Dpt/2300002M23Rik/Pxdn/Mamdc2/Mfap4/Nav2/Tnxb/Mmp1a/Col4a6* |
| GO:0004497 | monooxygenase activity | 2.39 | *Cyp2j11/Cyp1a1/Cyp1a2/Cyp1b1/Cyp2c38/Cyp2c39/Cyp2f2/Cyp4a10/Cyp4a14/Cyp4b1/Cyp51/Cyp7a1/Cyp8b1/Sqle/Mtrr/Tph2/Cyp2j13/Hpdl/Cyp2c54/Cyp2w1/Fmo2/Cyp4f14/Msmo1/Cyp4a31/Cyp2c66/Cyp2u1/Cyp4f18/Cyp2s1/Akr1c21* |
| GO:1901617 | organic hydroxy compound biosynthetic process | 2.33 | *Fdps/Adcyap1r1/Apoa4/Bmp6/Cyp51/Cyp7a1/Fdft1/Fgf15/Fgf2/Gfi1/Hmgcr/Hmgcs2/Hsd17b1/Ifng/Fabp5/Ldhb/Lhcgr/Lss/Mas1/Mvk/Myo5a/Nsdhl/Pla2g5/Mvd/Ptk2b/Nr1h4/Snai2/Sptlc2/Hmgcs1/Tph2/Nr1d1/Trpc1/Sc5d/Npc1l1/Dkk3/Nus1/Msmo1/Isyna1/Dhcr24* |
| GO:0031012 | extracellular matrix | 2.31 | *Spon2/Adamts9/Gpc5/Ltbp4/Alpl/Prelp/Col11a1/Col12a1/Col14a1/Col4a3/Col4a4/Col6a1/Col8a1/Hapln1/Vcan/Ncan/Epyc/Ecm1/Sparcl1/Col26a1/Fbln1/Fbn2/Fgf9/Fgfr2/Hsp90aa1/Igf1/Ihh/Lama2/Lama4/Lect1/Mmp2/Mmp24/Mmp7/Mmp8/Mmp9/Ogn/Serpine1/Reln/Apcs/Serpinf1/Slpi/Sparc/Thsd4/Efemp1/Adamts2/Thbs1/Thbs2/Timp3/Clec3b/Wnt10a/Wnt10b/Wnt2/Sbspon/Megf9/Spon1/Fbln5/Gpc6/Frem2/Otoa/Egflam/Ltbp1/Optc/Adamts16/Emilin3/Abi3bp/Hapln4/Postn/Hmcn1/Cpxm2/Htra1/Dpt/2300002M23Rik/Pxdn/Mamdc2/Fgfbp3/Mfap4/Nav2/Tnxb/Mmp1a/Col4a6* |
| GO:0008202 | steroid metabolic process | 2.30 | *Pcsk9/Fdps/Akr1c20/Apoa4/App/Bmp6/Serpina6/Cebpa/Cyp1a2/Cyp1b1/Cyp51/Cyp7a1/Fdft1/Fgf15/Gfi1/Hmgcr/Hmgcs2/Hsd11b1/Hsd17b1/Hsd17b4/Hsd3b5/Ifng/Igf1/Il1a/Lhcgr/Lss/Mvk/Nsdhl/Mvd/Nr1h4/Scd1/Snai2/Sqle/Sult1e1/Hmgcs1/Nr1d1/Pde8b/Sc5d/Npc1l1/Dhrs9/Slco1a1/Slco1a6/Dkk3/Sult2b1/Atp8b1/Cubn/Hsd17b14/Msmo1/Serpina12/Dhcr24/Akr1c21/Akr1c6* |
| GO:0005506 | iron ion binding | 2.23 | *Alox5/Cyp1a1/Cyp1a2/Cyp1b1/Cyp2c38/Cyp2c39/Cyp2f2/Cyp4a10/Cyp4a14/Cyp4b1/Cyp51/Cyp7a1/Cyp8b1/Heph/Lcn2/Ltf/Scd1/Mtrr/Tph2/Sc5d/Nt5e/Fa2h/Cyp2c54/Cyp2w1/Cyp4f14/Msmo1/Cyp2u1/Aox3/Cyp4f18/Cyp2s1* |
| GO:0060326 | cell chemotaxis | 2.13 | *Adam8/C5ar1/Cxcr2/Cxcr3/Ccr2/Csf3r/Fgf2/Cxcl1/Hspb1/Cxcl10/Ifng/Il12a/Nrp1/Pde4b/Pik3c2a/Prkca/Prkcq/Serpine1/Ptk2b/Ptpro/Saa3/Saa4/Ccl3/Ccl4/Cxcl2/Cxcl5/Snai2/Thbs1/Vcam1/Ffar2/Dock4/Tnfsf18/Ephb1/Cxcl13/Stap1/Trem1/Pik3cb/Il23a* |
| GO:0050795 | regulation of behavior | 2.07 | *Arrdc3/C5ar1/Casr/Cxcr2/Cxcr3/Ccr2/Dlg4/Fgf2/Cxcl1/Hspb1/Cxcl10/Il12a/Il6/Itga2/Kdr/Cntnap4/Nrp1/Prkca/Serpine1/Ccl21a/Ptger3/Ptger4/Ptk2b/Ptpn2/Reln/Ccl4/Cxcl2/Cxcl5/St6gal1/Snai2/Stx3/Thbs1/Tnfsf18/Uts2/Plxna4/Cxcl13/Stap1/Retn/Ghrl/Sgip1/Il23a* |
| GO:0040017 | positive regulation of locomotion | 2.04 | *Fam110c/Adam8/Adora3/Adra2a/C5ar1/Casr/Cxcr2/Cxcr3/Ccr2/Cpeb1/Cyp1b1/Egr1/Erbb4/Fbln1/Fgf2/Gcnt2/Cxcl1/Hspa5/Hspb1/Cxcl10/Ifng/Igf1/Il12a/Il1a/Itga2/Kdr/Mmp2/Mmp9/Nrp1/Prkca/Serpine1/Ccl21a/Ptger3/Ptger4/Ptk2b/Reln/Ccl20/Ccl4/Cxcl2/Cxcl5/Slc8a1/Snai2/Sparc/Stx3/Tac1/Tfap2a/Tdgf1/Tek/Thbs1/Tnfaip6/Twist1/Epb41l5/Arhgef39/Kif20b/Tnfsf18/Tlr2/Uts2/Glipr2/Tmprss2/Postn/Cxcl13/Rapgef4/Retn/Lrrc16a/Rps6kb1/Lrrc15/Clasp1/Slc26a5/Cemip/Il23a/Mmp1a* |
| GO:0014069 | postsynaptic density | 2.02 | *Ank2/Add2/Axin2/Cpeb1/Dab1/Dlg4/Erbb4/Grid2/Grin2b/Lrp8/Map1a/Map4/Mapt/Neurl1a/Ntrk2/Palm/Pde4b/Ptk2b/Exoc4/Sptbn1/Ablim1/Klhl17/Erc2/Itga8/Ppp1r9a/Shank1/Syn3/Adgrl1/Nefh/Gria3/Srcin1/Clstn2/Clstn1/Cpeb4/Pdzd2* |
| GO:0032103 | positive regulation of response to external stimulus | 2.02 | *Ace/Adam8/Adora3/Bmp6/C5ar1/Casr/Cxcr2/Cxcr3/Ccr2/Fcer1a/Fgf2/Cxcl1/Hspb1/Cxcl10/Il12a/Il6/Irg1/Itga2/Kdr/Nrp1/Pafah1b2/Prkca/Serpine1/Ccl21a/Ptger3/Ptger4/Ptk2b/Ccl3/Ccl4/Cxcl2/Cxcl5/Snai2/Stx3/Tac1/Thbs1/Ffar2/Tnfsf18/Tlr2/Scarf1/Cxcl13/Ghrl/Trim13/Il23a* |
| GO:0006935 | chemotaxis | 2.01 | *Mycbp2/Adam8/Alcam/App/Bdnf/Cxcr5/C5ar1/Casr/Cdh4/Cxcr2/Cxcr3/Ccr2/Ackr3/Csf3r/Dlx5/Egr2/Fgf2/Gas1/Gbx2/Ptgdr2/Cxcl1/Foxg1/Hspb1/Cxcl10/Ifng/Il12a/Itga2/Kdr/Kif5c/Lama2/Lhx2/Nrp1/Pde4b/Pik3c2a/Prkca/Prkcq/Serpine1/Ccl21a/Ptk2b/Ptpn2/Ptprm/Ptpro/Robo3/Reln/Saa3/Saa4/Ccl20/Ccl3/Ccl4/Ccl7/Cxcl2/Cxcl5/Sema5b/St6gal1/Slit3/Snai2/Stx3/Thbs1/Vax1/Vcam1/Rnf165/Ablim1/Cxcl17/Ffar2/Dock4/Tnfsf18/Plxna4/Dpysl4/Ephb1/Flrt2/Prok2/Cxcl13/Stap1/Bcl11b/Trem1/Ackr2/Cmtm5/Pik3cb/Il23a* |
| GO:0051272 | positive regulation of cellular component movement | 2.01 | *Fam110c/Adam8/Adora3/Adra2a/Bcl6/C5ar1/Cxcr2/Ccr2/Cpeb1/Cyp1b1/Egr1/Erbb4/Fbln1/Fgf2/Gcnt2/Cxcl1/Hspa5/Hspb1/Cxcl10/Ifng/Igf1/Il12a/Il1a/Itga2/Kdr/Mmp2/Mmp9/Nrp1/Prkca/Serpine1/Ccl21a/Ptger3/Ptger4/Ptk2b/Reln/Ccl20/Ccl4/Cxcl2/Cxcl5/Slc8a1/Snai2/Sparc/Tac1/Tfap2a/Tdgf1/Tek/Thbs1/Tnfaip6/Twist1/Epb41l5/Arhgef39/Kif20b/Tnfsf18/Tlr2/Uts2/Glipr2/Postn/Cxcl13/Rapgef4/Retn/Lrrc16a/Rps6kb1/Lrrc15/Clasp1/Slc26a5/Cemip/Il23a/Mmp1a* |
| GO:0042330 | taxis | 2.01 | *Mycbp2/Adam8/Alcam/App/Bdnf/Cxcr5/C5ar1/Casr/Cdh4/Cxcr2/Cxcr3/Ccr2/Ackr3/Csf3r/Dlx5/Egr2/Fgf2/Gas1/Gbx2/Ptgdr2/Cxcl1/Foxg1/Hspb1/Cxcl10/Ifng/Il12a/Itga2/Kdr/Kif5c/Lama2/Lhx2/Nrp1/Pde4b/Pik3c2a/Prkca/Prkcq/Serpine1/Ccl21a/Ptk2b/Ptpn2/Ptprm/Ptpro/Robo3/Reln/Saa3/Saa4/Ccl20/Ccl3/Ccl4/Ccl7/Cxcl2/Cxcl5/Sema5b/St6gal1/Slit3/Snai2/Stx3/Thbs1/Vax1/Vcam1/Rnf165/Ablim1/Cxcl17/Ffar2/Dock4/Tnfsf18/Plxna4/Dpysl4/Ephb1/Flrt2/Prok2/Cxcl13/Stap1/Bcl11b/Trem1/Ackr2/Cmtm5/Pik3cb/Il23a* |
| GO:0006954 | inflammatory response | 1.93 | *Ace/Adam8/Adora3/Adra2a/Agtr1b/Alox5/Bcl6/Bdkrb2/Camk4/Cebpb/Chil1/Cxcr2/Cxcr3/Ccr2/Cr1l/Cd55/Ackr1/Ecm1/Ephb6/Mecom/Fcer1a/Ms4a2/Cxcl1/Hmox1/Ier3/Cxcl10/Il1a/Il1rn/Il6/Irg1/Itga2/Itgb6/Itih4/Klk1b1/Mas1/Mvk/Ncf1/Orm1/Orm2/Reg3b/Per1/Pik3c2a/Prkca/Prkcq/Serpine1/Ptger3/Ptger4/Ptpn2/Rel/Saa3/Saa4/Ccl20/Ccl3/Ccl4/Ccl7/Cxcl2/Cxcl5/Serpinf1/Il23r/Tac1/Tff2/Thbs1/Tnfaip3/Tnfaip6/Tyro3/A2m/Ffar2/Mgll/Nt5e/Tnfsf18/Tlr2/Nlrp4a/Il19/Cd59b/Calcrl/Cxcl13/Ghrl/Ackr2/Duoxa2/Wfdc1/Tspan2/Pik3cb/Ak7/Nfkbiz/Il23a/Pik3ap1* |

*Supplementary Figure S1. The effect of curcumin on individual bile acids after ANIT administration in both WT and FXRKO mice.* Serum CA, TCA, TDCA, TCDCA, αMCA, βMCA, ωMCA, TαMCA, TβMCA and TωMCA levels were quantified by LC/MS in both WT and FXRKO mice. Data are presented as mean ± SD. **p*<0.05.

*Supplementary Figure S2. Cluster analysis of the protective effect of curcumin against ANIT.* The protective effect of curcumin against ANIT was studied by cluster analysis based on the selected genes which contributed most to cholestasis. Selected genes were visualized in a heatmap plot. The colors on the heatmap correspond to the contents of bile acids Red represents the increase, while green represents the decrease. Hierarchical clustering separates X axis of heatmap represents different groups, while Y axis stands for different genes .

***Supplementary Figure S1.***

*
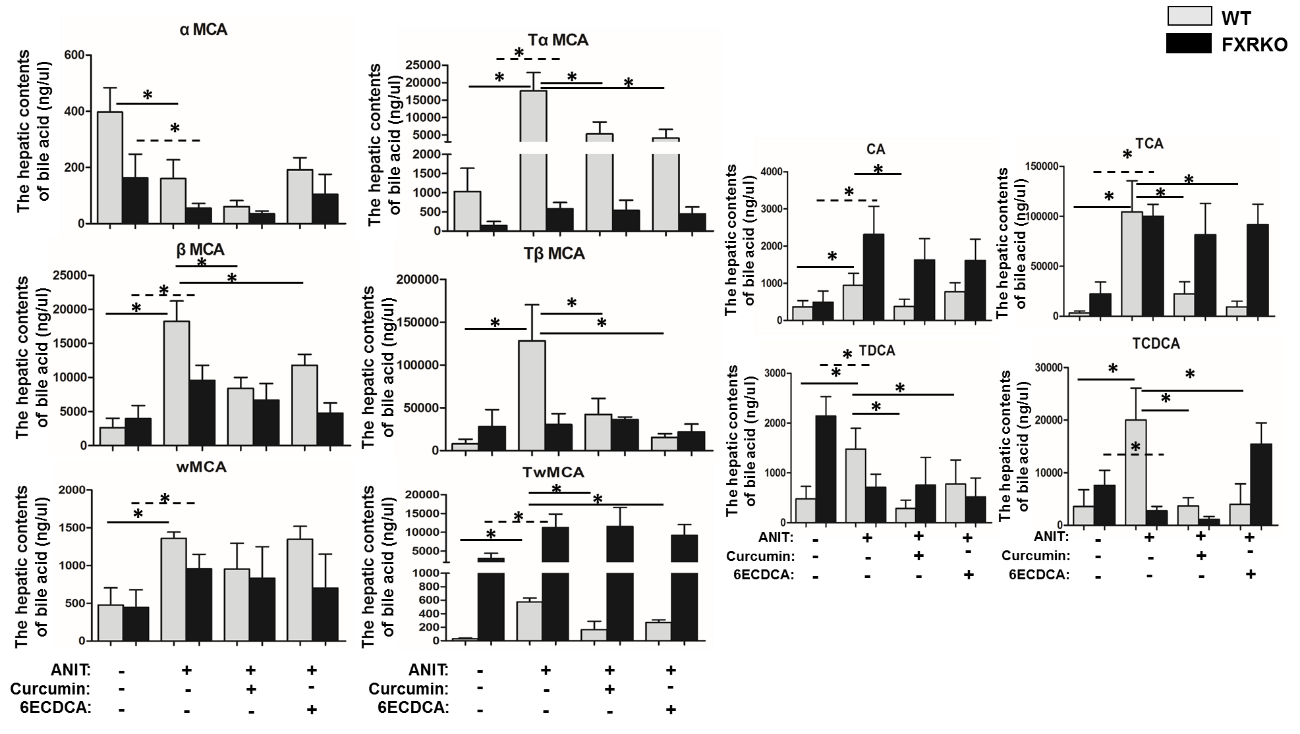
*

***Supplementary Figure S2.***

*
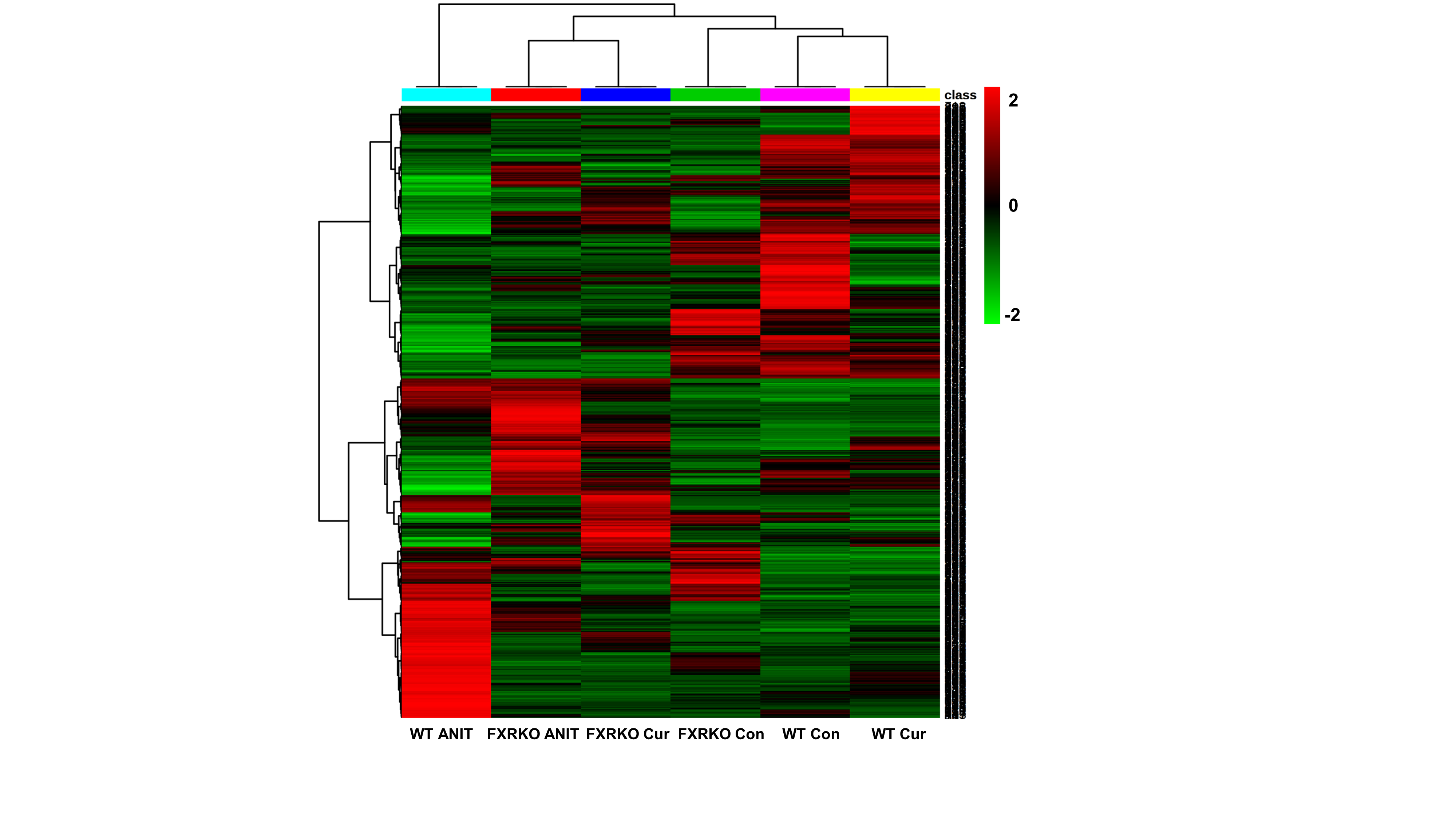
*
